# Photoperiod-driven gene regulation in the brain of *Chirostoma estor*: endocrine, metabolic, and epigenetic insights

**DOI:** 10.3389/fendo.2026.1818535

**Published:** 2026-05-01

**Authors:** Mitzi Ernestina Juárez-Gutiérrez, Valeria Colin-Orozco, Zoraya Palomera-Sánchez, Pamela Navarrete-Ramírez, Carlos Cristian Martínez-Chávez

**Affiliations:** 1 Laboratorio Nacional de Nutrigenómica y Microbiómica Digestiva Animal, Instituto de Investigaciones Agropecuarias y Forestales, Universidad Michoacana de San Nicolás de Hidalgo, Morelia, Michoacán, Mexico; 2 Facultad de Medicina Veterinaria y Zootecnia, Universidad Michoacana de San Nicolás de Hidalgo, Morelia, Michoacán, Mexico; 3 Secretaria de Ciencia, Humanidades, Tecnología e Innovación (SECIHTI), Laboratorio Nacional de Nutrigenómica y Microbiómica Digestiva Animal, Instituto de Investigaciones Agropecuarias y Forestales, Universidad Michoacana de San Nicolás de Hidalgo, Morelia, Michoacán, Mexico

**Keywords:** appetite regulation, continuous illumination, epigenetic remodeling, fish neuroendocrinology, ROS responses, somatotropic axis

## Abstract

**Introduction:**

Aquatic light environments are increasingly altered by human activity, yet the endocrine and molecular mechanisms enabling fish to cope with extreme photoperiods remain poorly understood. This study investigates neuroendocrine and transcriptomic responses to continuous illumination in *Chirostoma estor*.

**Methods:**

We conducted an *in silico*, hypothesis-driven reanalysis of a juvenile brain transcriptome obtained at 12 weeks post-hatching under continuous light (LL) or a control 12 h light: 12 h dark photoperiod. The analysis focused on genes associated with somatic growth, thyroid signaling, oxidative balance, appetite regulation, and epigenetic processes.

**Results:**

Continuous illumination induced a transcriptional profile consistent with enhanced growth signaling sensitivity, including increased expression of growth hormone receptor and insulin-like growth factor pathway components, alongside reduced growth hormone transcripts and somatostatin receptor expression. Thyroid signaling showed reciprocal regulation of receptor isoforms, suggesting a growth-permissive mechanism. LL also upregulated antioxidant and redox-buffering genes, including mitochondrial uncoupling protein 2. In contrast, stress-axis regulators such as corticotropin-releasing factor and glucocorticoid receptor were more highly expressed under the control photoperiod. Epigenetic and nuclear architecture regulators displayed coordinated changes, indicating chromatin remodeling linked to selective pathway activation. Appetite-related genes showed mixed regulation patterns.

**Discussion:**

These findings support a model in which continuous illumination promotes an alternative neuroendocrine state integrating growth-axis rewiring, oxidative stress protection, and epigenetic remodeling. This plastic response provides mechanistic insight into photoperiod-driven adaptation, with implications for understanding environmental light disruption and optimizing photoperiod management in aquaculture.

## 1 Introduction

Endocrine systems in fish are a central interface through which environmental information is converted into physiological action, coordinating feeding behavior, metabolism, growth, and the allocation of energy among tissues (1). Environmental cues such as photoperiod, temperature, oxygen availability, and nutrient supply are integrated by neuroendocrine networks that connect the brain, pituitary, liver, and peripheral organs, enabling coordinated whole-body responses (2). This integration relies on conserved hormonal axes, including the growth hormone-insulin-like growth factor axis, the hypothalamic-pituitary-thyroid axis, and neuropeptide systems that regulate appetite and energy balance, among others (1, 3).

Alongside thermal change and oxygen stress, aquatic ecosystems are undergoing major shifts in their light environment, driven by land use change, altered hydrology, and climate-related processes (4, 5). Light is a dominant Zeitgeber for biological clocks and synchronizes circadian and seasonal rhythms through neuroendocrine pathways that regulate melatonin secretion, reproductive timing, and metabolic organization (3, 6). However, the natural structure of daily and seasonal light signals is increasingly distorted at both global and local scales (4).

A prominent driver of changing underwater light climates is freshwater browning, often linked to rising inputs of terrestrially derived dissolved organic matter, which reduces light penetration and alters spectral quality (4, 7). Long-term browning can reshape vertical habitat gradients and food web structure, effectively changing the photic information available to aquatic organisms (8). In parallel, artificial light at night is expanding across coastal zones, rivers, and lakes, increasing nocturnal illumination and modifying spectral composition in ways that can suppress or shift melatonin rhythms and disrupt circadian organization (9, 10).

From an endocrine perspective, these changes can disrupt the alignment between internal biological rhythms and external environmental cues, with downstream effects on fitness, population dynamics, and ecosystem function (8, 10). Determining when endocrine systems can compensate for altered light cues, and when compensation fails, is therefore essential for predicting resilience under accelerating environmental change (1, 4).

Photoperiodic information is transduced into endocrine signals that regulate growth and metabolism at multiple levels. In fish, light cues influence hypothalamic signaling, pituitary hormone release, peripheral tissue sensitivity, and downstream metabolic pathways (1, 6). Photoperiod manipulation has long been used in aquaculture to extend feeding windows and reshape endocrine rhythms, but responses to prolonged or continuous illumination are strongly species and context-dependent (9, 11). While growth enhancement is reported for most teleosts, constant illumination can also be associated with physiological stress responses and welfare concerns in others, emphasizing the need to identify mechanisms that separate adaptive cueing from chronic disruption (1, 9).

Within this context, *Chirostoma estor* (Atherinopsidae) offers a powerful model for examining endocrine and metabolic plasticity under extreme photoperiod conditions. Individuals reared under continuous illumination (LL) from fertilization develop a rapid growth phenotype, attaining substantially greater body mass and length than conspecifics reared under a 12L:12D (LD; light:dark) photoperiod, without reductions in condition factor. This phenotype is accompanied by persistently low whole-body cortisol, suggesting that continuous light does not trigger chronic activation of the hypothalamic-pituitary-interrenal axis but instead supports an alternative endocrine state compatible with enhanced growth (12). More broadly, organisms that develop without a day-night cycle provide a useful biological framework for considering how life can function under atypical photic regimes, a question that also intersects with astrobiology and the assessment of habitability under prolonged illumination or darkness (13, 14).

In this work, brain transcriptomic data from juvenile *C. estor* reared under contrasting photoperiods (12) were used to assess how continuous illumination reshapes endocrine and metabolic regulation. The expression levels of genes linked to somatic growth, antioxidant and genome stability pathways, neuroendocrine regulation of appetite and energy balance were analyzed *in silico* to support an integrated endocrine view of photoperiod as a key modulator of feeding and metabolism in fish.

## 2 Materials and methods

### 2.1 Original experiment and dataset

The original experiment (12) was carried out at the Laboratorio Nacional de Nutrigenómica y Microbiómica Digestiva Animal (LANMDA-IIAF), Universidad Michoacana de San Nicolás de Hidalgo, Morelia, México. In that experiment, *Chirostoma estor* were reared from manual fertilization under controlled conditions at 20 ± 1 °C and exposed to two photoperiod regimes: a control light-dark (LD) cycle of 12 h light and 12 h dark, and continuous illumination (LL) of 24 h light and 0 h dark. Light was provided by broad-spectrum commercial cold white T8-LED lamps (6500 K; 26.14 W m^-2^; Megaluz mod. TDL001, China). During early development, larvae were fed *ad libitum* with live rotifers (*Brachionus plicatilis*) and *Artemia franciscana* up to 4 weeks after hatching (wah), after which a species-specific formulated diet was supplied *ad libitum* four times during the day and four times during the night using automatic feeders (ZW82, BOYU^®^, China) in both treatments (12). At 12 weeks post-hatching, brain tissue was collected, placed in RNAlater^®^ (Invitrogen, Thermo Fisher Scientific, Waltham, MA, USA), and stored at −80 °C until downstream processing (12).

Total RNA was extracted from brain samples and used to construct RNA sequencing libraries. Libraries were sequenced using Genome Analyzer IIx (Illumina, San Diego, CA, USA). This transcriptome was previously validated by Corona-Herrera et al. (15).

In Corona-Herrera et al. (12) a differential gene expression analysis (p-value ≤ 0.05 and a LogFold change ≥ 2) was performed between LD and LL treatments using six samples. Transcripts were indexed, aligned, and mapped against the reference transcriptome of *C. estor* (PRJNA401428) with Bowtie2 (16). Transcript abundance was estimated using RSEM (17) and eXpress (18). The raw count table was used as input for differential expression analysis with EdgeR/Bioconductor (19, 20), applying trimmed mean of M-values (TMM) normalization. Gene Ontology (GO) terms were obtained via the Panther classification system and NCBI database to explore associated biological processes and metabolic pathways (12). The functional transcriptome annotation report from (12) served as the input data for the present work.

### 2.2 Bioinformatic reanalyses

In the present work, the original annotation report by Corona-Herrera et al. (12) was reanalyzed to identify additional biologically relevant genes associated with growth, oxidative stress, epigenetic regulation, and appetite responses in *C. estor* that may have been overlooked under the more stringent filtering criteria of the initial study. To this end, a structured literature and bioinformatic survey was conducted to define candidate genes, prioritizing those with well-established roles in teleosts and other vertebrates, particularly within conserved endocrine and metabolic pathways such as the GH-IGF axis, thyroid signaling, redox systems, and neuropeptide regulation. Gene identities and functional annotations were validated using public databases, including NCBI and the PANTHER classification system. The resulting curated gene set was then used to screen the original brain transcriptome annotation dataset, enabling the identification of corresponding transcripts in juvenile *C. estor*. These transcripts were subsequently analyzed for differential expression between the LD and LL treatments using updated statistical criteria (p < 0.05; LogFold change = 1). This comparison was performed using the transcript abundance matrices generated during the bioinformatics processing stage and the IDEAMEX platform (Integrated Differential Expression Analysis MultiEXperiment; accessed 16 January 2025; https://www.uusmb.unam.mx/ideamex2/index.php) (21). This workflow enabled visualization and comparison of expression differences for the selected genes between the two photoperiod conditions.

## 3 Results

Growth-associated genes identified through functional annotation are summarized in **Table 1**. Differential expression analysis revealed a clear segregation of transcripts into inhibitory and promotive roles in growth, with the latter showing a consistent bias toward upregulation under LL.

**Table 1:** Transcript expression level of growth-related genes between the two treatments: light/dark (LD, 12:12 h) and continuous light (LL, 24 h light).

Genes that inhibit growth						
Gene	Name	Transcript		Expression		Treatment with higher expression
ING	Inhibitor of growth family members	ING1	Inhibitor of growth protein 1	N/E	N/E	N/E
		ING2	Inhibitor of growth protein 2	↓ LL	↑ LD	LD
		ING3	Inhibitor of growth protein 3	↑ LL	↓ LD	LL
		ING4	Inhibitor of growth protein 4	↑ LL	↓ LD	LL
		ING5	Inhibitor of growth protein 5	↓ LL	↑ LD	LD
PTEN	Phosphatase and tensin homolog	N/F	N/F	N/E	N/E	N/E
SSR	Somatostatin	SSTR2	Somatostatin receptor type 2	↓LL	↑ LD	LD
		SSTR5	Somatostatin receptor type 5	↓ LL	↑ LD	LD
NCoR1	Nuclear receptor corepressor 1	N/F	N/F	N/E	N/E	N/E
GDF-8	Myostatin (Growth differentiation factor 8)	GDF-8	Growth differentiation factor 8	N/E	N/E	N/E
PACA	Pituitary adenylate cyclase- activating polypeptide	PACA	Pituitary adenylate cyclase- activating polypeptide	N/E	N/E	N/E
GR	Glucocorticoid receptor	N/F	N/F	N/E	N/E	N/E
MyoD	Myogenic differentiation	MDFI	MyoD family inhibitor	N/E	N/E	N/E
Genes that promote growth						
Gene	Name	Transcript		Expression		Treatment with higher expression
GH	Growth hormone	GH	Growth hormone	↓ LL	↑ LD	LD
GHR	Growth hormone receptor	GHR	Growth hormone receptor	↑ LL	↓ LD	LL
IGF1	Insulin-like growth factor 1	IGF2	Insulin-like growth factor 2	↑ LL	↓ LD	LL
IGF1R	Insulin-like growth factor 1 receptor	IGF1R	Insulin-like growth factor 1 receptor	↑ LL	↓ LD	LL
IGFBP	Insuline-like growth factor-binding protein	IGFBP1	Insuline-like growth factor-binding protein 1	↓ LL	↑ LD	LD
		IGFBP2A	Insulin-like growth factor-binding protein 2-A	↓ LL	↑ LD	LD
		IGFBP2B	Insulin-like growth factor-binding protein 2-B	N/E	N/E	N/E
		IGFBP3	Insulin-like growth factor-binding protein 3	↓ LL	↑ LD	LD
		IGFBP5	Insulin-like growth factor-binding protein 5	↓ LL	↑ LD	LD
		IGFBP6	Insulin-like growth factor-binding protein 6	N/E	N/E	N/E
		IGFBP7	Insulin-like growth factor-binding protein 7	↑ LL	↓ LD	LL
mTOR	Mechanistic target of rapamycin	N/F	N/F	N/E	N/E	N/E
Myf5	Myogenic factor 5	Myf5	Myogenic factor 5	N/E	N/E	N/E
MyoD	Myogenic differentiation 1	N/F	N/F	N/E	N/E	N/E
TSH	Thyroid-stimulating hormone	N/F	N/F	N/E	N/E	N/E
TPO	Thyroid peroxidase	N/F	N/F	N/E	N/E	N/E
TR	Thyroid hormone receptor	THRA	Thyroid hormone receptor alpha	↓ LL	↑ LD	LD
		THRB	Thyroid hormone receptor beta	↑ LL	↓ LD	LL
DIO	Iodothyronine deiodinase	N/F	N/F	N/E	N/E	N/E
MyHC	Myosin heavy chains	MYSS	Myosin heavy chain, fast skeletal muscle	N/E	N/E	N/E
		MYSA	Myosin heavy chain, muscle	N/E	N/E	N/E
		MYSU	Myosin heavy chain, embryonic smooth muscle	↓ LL	↑ LD	LD

An upward arrow (↑) represents the treatment with the highest gene expression, and a downward arrow (↓) represents the treatment with the lowest gene expression (p<0.05; LogFold change = 1). N/E, no expression; N/F, not found.

Continuous illumination produced distinct and directionally consistent changes in the expression of growth-related and endocrine genes. Several transcripts with established growth-inhibitory roles were significantly downregulated under LL, including somatostatin receptor types 2 and 5 (SSTR2, SSTR5) and inhibitor of growth family members (ING2 and ING5). In contrast to these downregulated inhibitory transcripts, ING3 and ING4 were significantly upregulated under LL.

Conversely, multiple growth-promoting and related genes displayed higher expression under LL. These included the growth hormone receptor (GHR), insulin-like growth factor 2 (IGF2), and the insulin-like growth factor 1 receptor (IGF1R). Notably, this occurred despite a significant downregulation of growth hormone (GH) transcripts under LL conditions. Within the insulin-like growth factor-binding protein family, most members (IGFBP1, IGFBP2A, IGFBP3, and IGFBP5) were downregulated under LL, whereas IGFBP7 exhibited significant upregulation.

Photoperiod-dependent regulation was also evident in the thyroid hormone signaling pathway. Transcripts encoding thyroid hormone receptor alpha (THRA) were downregulated under LL, while thyroid hormone receptor beta (THRB) was significantly upregulated. Finally, myosin heavy chain (MyHC) transcripts encoding embryonic smooth muscle myosin heavy chain (MYSU) were significantly downregulated in LL-treated fish.

Several genes annotated as growth-inhibitory or growth-promoting in teleosts were either not expressed or not detected in the brain of *C. estor* (**Table 1**).

**Table 2** shows genes related to oxidative stress that were differentially expressed in the transcriptome between the two treatments. Exposure to LL was associated with a significant upregulation of multiple antioxidant defense genes, including superoxide dismutase isoforms (SODC, SODE), glutathione reductase (GSR), glutathione S-transferases (GSTM3, MGST1, MGST3), and glutathione peroxidases (GPX1, GPX7). While some antioxidant transcripts were also elevated under the light/dark (LD) condition, their expression levels were consistently higher under LL.

**Table 2:** Transcript expression level of genes related to oxidative stress between the two treatments: light/dark (LD, 12:12 h) and continuous light (LL, 24 h light).

Genes associated with oxidative stress						
Gene	Name	Transcript		Expression		Treatment with higher expression
CAT	Catalase	CATA	Catalase	↑ LD	↓ LL	LD
SOD	Superoxide dismutase	CCS	Copper chaperone for superoxide dismutase	↑ LD	↓ LL	LD
		SODC	Superoxide dismutase [Cu-Zn]	↓ LD	↑ LL	LL
		SODM	Superoxide dismutase [Mn], mitochondrial	↑ LD	↓ LL	LD
		SODE	Extracellular superoxide dismutase [Cu-Zn]	↓ LD	↑ LL	LL
GSR	Glutathione reductase	GSHR	Glutathione reductase, mitochondrial	↓ LD	↑ LL	LL
GST	Glutathione S-transferase	GSTM3	Glutathione S-transferase Mu 3	↓ LD	↑ LL	LL
		GSTO1	Glutathione S-transferase omega-1	↑ LD	↓ LL	LD
		GSTCD	Glutathione S-transferase C-terminal domain-containing protein	↑ LD	↓ LL	LD
		GSTA4	Glutathione S-transferase A4	↑ LD	↓ LL	LD
		MGST1	Microsomal glutathione S-transferase 1	↓ LD	↑ LL	LL
		MGST3	Microsomal glutathione S-transferase 3	↑ LD	↓ LL	LD
		MGST3	Microsomal glutathione S-transferase 3	↓ LD	↑ LL	LL
GPX	Glutathione peroxidase	GPX7	Glutathione peroxidase 7	↓ LD	↑ LL	LL
		GPX4	Phospholipid hydroperoxide glutathione peroxidase, mitochondrial	↑ LD	↓ LL	LD
		GPX1	Glutathione peroxidase 1	↑ LD	↓ LL	LD
		GPX1	Glutathione peroxidase 1	↓ LD	↑ LL	LL
UCP	Uncopling protein	UCP2	Mitochondrial uncoupling protein 2	↓ LD	↑ LL	LL
CRF	Corticotropin-releasing factor	CRHBP	Corticotropin-releasing factor-binding protein	↑ LD	↓ LL	LD
		CRFR2	Corticotropin-releasing factor receptor 2	↑ LD	↓ LL	LD
GCR	Glucocorticoid receptor	GCR	Glucocorticoid receptor	↑ LD	↓ LL	LD

An upward arrow (↑) represents the treatment with the highest gene expression, and a downward arrow (↓) represents the treatment with the lowest gene expression (p<0.05; LogFold change = 1).

In addition, the mitochondrial uncoupling protein 2 (UCP2) transcript was significantly upregulated in LL-treated fish. In contrast to this LL-associated upregulation, transcripts encoding corticotropin-releasing factor (CRF) and the glucocorticoid receptor (GR) showed significantly higher expression under LD conditions, indicating a photoperiod-dependent divergence in oxidative stress and stress-axis-related gene regulation.

In juvenile *C. estor*, comparisons between photoperiods revealed consistent modulation of epigenetic regulators and genes involved in nuclear organization and genome surveillance (**Table 3**). Within the transcriptional repression group, LL was associated with higher expression of HDAC4 (histone deacetylase 4), HDAC7 (histone deacetylase 7) and KDM5B (lysine demethylase 5B), whereas LD was associated with higher expression of KMT2C (lysine methyltransferase 2C), p300 (EP300 lysine acetyltransferase) and KDM6A (lysine demethylase 6A). In contrast, among genes linked to transcriptional activation, LL exhibited increased expression of the histone variant H3.3 and the acetylation readers BRD1, BRD3, and BRD4 (bromodomain containing 1, 3 and 4), while histone methyltransferase EZH2 (enhancer of zeste 2 polycomb repressive complex 2 subunit) and AGO3 (argonaute RISC catalytic component 3) were more highly expressed under LD. Regarding nuclear architecture and the DNA damage response, CTCF (CCCTC-binding factor) was upregulated in LL, whereas the DNA damage sensor ATM (ATM serine/threonine kinase) showed higher expression in LD. **Table 4** summarizes genes involved in appetite regulation in teleost fish, with transcripts classified according to established functions as either orexigenic (appetite-stimulating) or anorexigenic (appetite-inhibiting).

**Table 3:** Transcript expression level of genes related to epigenetic marks between the two treatments: light/dark (LD, 12:12 h) and continuous light (LL, 24 h light).

Transcriptional repression				
Gene	Name	Expression		Treatment with higher expression
HDAC7	Histone deacetylase 7	↓ LD	↑ LL	LL
HDAC4	Histone deacetylase 4	↓ LD	↑ LL	LL
KMT2C	Lysine methyltransferase 2C	↑ LD	↓ LL	LD
p300	EP300 lysine acetyltransferase	↑ LD	↓ LL	LD
KDM6A	Lysine demethylase 6A	↑ LD	↓ LL	LD
KDM5B	Lysine demethylase 5B	↓ LD	↑ LL	LL
Transcriptional activation				
H3.3	Histone variant	↓ LD	↑ LL	LL
EZH2	Enhancer of zeste 2 polycomb repressive complex 2 subunit	↑ LD	↓ LL	LD
AGO3	Argonaute RISC catalytic component 3	↑ LD	↓ LL	LD
BRD1	Bromodomain containing 1	↓ LD	↑ LL	LL
BDR3	Bromodomain containing 3	↓ LD	↑ LL	LL
BDR4	Bromodomain containing 4	↓ LD	↑ LL	LL
Nuclear architecture				
CTCF	CCCTC binding factor	↓ LD	↑ LL	LL
DNA sensor damage				
ATM	ATM serine/threonine kinase	↑ LD	↓ LL	LD

An upward arrow (↑) represents the treatment with the highest gene expression, and a downward arrow (↓) represents the treatment with the lowest gene expression (p<0.05; LogFold change = 1).

**Table 4:** Transcript expression level of genes related to appetite control between the two treatments: light/dark (LD, 12:12 h) and continuous light (LL, 24 h light).

Orexigenic genes (stimulate appetite)						
Gene	Name	Transcript		Expression		Treatment with higher expression
APJ	Apelin	APJA	Apelin receptor A	↑ LD	↓ LL	LD
		APEL	Apelin	↓ LD	↑ LL	LL
		APJB	Apelin receptor B	N/E	N/E	N/E
GHRL	Ghrelin	N/F	N/F	N/E	N/E	N/E
OX	Orexin	N/F	N/F	N/E	N/E	N/E
GAL	Galanin	GALA	Galanin receptor type 1	N/E	N/E	N/E
		GALR1	Galanin peptides	N/E	N/E	N/E
SgII	Secretoneurin (secretogranin precursor)	SCG1	Secretogranin-1	↑ LD	↓ LL	LD
MCH	Melanin-concentrating hormone	MCH	Pro-MCH	↑ LD	↓ LL	LD
AGRP	Agouti-related protein	AGRP	Agouti-related protein	↑ LD	↓ LL	LD
NPY	Neuropeptide Y	PY	Peptide Y	N/E	N/E	N/E
		NPY	Pro-neuropeptide Y	N/E	N/E	N/E
Anorexigenic genes (inhibit appetite)						
Gene	Name	Transcript		Expression		Treatment with higher expression
LEP	Leptin	LEPR	Leptin receptor	N/E	N/E	N/E
NEFA-1/NUCB2	Nesfatin/Nucleobindin	NUCB2	Nucleobindin-2	↑ LD	↓ LL	LD
		NUCB1	Nucleobindin-1	N/E	N/E	N/E
SPX	Spexin	SPXN	Spexin	↓ LD	↑ LL	LL
IAPP	Amylin	N/F	N/F	N/E	N/E	N/E
CCK	Cholecystokinin	CCKAR	Cholecystokinin	↑ LD	↓ LL	LD
		CCKN	Cholecystokinin receptor type A	N/E	N/E	N/E
GNRH	Gonadotropin-releasing hormone	N/F	N/F	N/E	N/E	N/E
MSH	Melanocyte-stimulating hormone	N/F	N/F	N/E	N/E	N/E
NMU	Neuromedin	NMU	Neuromedin-U receptor 2	N/E	N/E	N/E
		NMU	Neuromedin-U	N/E	N/E	N/E
		NMS	Neuromedin-S	N/E	N/E	N/E
		NMB	Neuromedin-B	N/E	N/E	N/E
ODN	Octadecaneuropeptide	N/F	N/F	N/E	N/E	N/E
AVT	Arginine-vasotocin	AVT	[Arg8]-vasotocin receptor	N/E	N/E	N/E
PRP	Prolactin	PRLR	Prolactin receptor	↑ LD	↓ LL	LD
		PRLR	Prolactin receptor	↓ LD	↑ LL	LL
CRF	Corticotropin-releasing factor	CRFR2	Corticotropin-releasing factor receptor 2	N/E	N/E	N/E
UCN	Urocortins	UCN3	Urocortin-3	N/E	N/E	N/E
CART	Cocaine- and amphetamine-regulated transcript	CART	Cocaine- and amphetamine-regulated transcript protein	N/E	N/E	N/E
POMC	Pro-opiomelanocortin	N/F	N/F	N/E	N/E	N/E
GRP/BBS	Gastrin-releasing peptide/Bombesin	N/F	N/F	N/E	N/E	N/E
GLP	Glucagon-like peptide	GLR	Glucagon receptor	N/E	N/E	N/E
		PACA	Glucagon family neuropeptides	↑ LD	↓ LL	LD
SP/NKA/NKB	Tachykinins: substance P/neurokinin A/neurokinin B	TKN1	Protachykinin	N/E	N/E	N/E
UI	Urotensins	N/F	N/F	N/E	N/E	N/E

An upward arrow (↑) represents the treatment with the highest gene expression, and a downward arrow (↓) represents the treatment with the lowest gene expression (p<0.05; LogFold change = 1). N/E, no expression; N/F, not found.

Photoperiod-dependent differences were also observed in genes associated with appetite regulation and energy balance. A substantial proportion of transcripts commonly described as orexigenic (e.g., ghrelin, orexin, galanin, NPY) or anorexigenic (e.g., leptin, amylin, gonadotropin-releasing hormone, melanocyte-stimulating hormone, neuromedin) were not detected in the brain transcriptome under either photoperiod condition (**Table 4**).

Among the orexigenic genes detected, only the apelin receptor A (APLNR) showed significant upregulation under LL. In contrast, other appetite-stimulating transcripts, including secretogranin (SCG1), melanin-concentrating hormone (MCH), and agouti-related protein (AGRP), were significantly downregulated under LL compared to LD. A similarly mixed pattern was observed for anorexigenic genes. Transcripts encoding spexin (SPX) and a prolactin receptor (PRLR) were significantly upregulated under LL, whereas nucleobindin 2 (NUCB2), cholecystokinin receptor (CCKAR), and a second prolactin receptor (PRLR) were downregulated under the same photoperiod.

## 4 Discussion

In the original study by Corona-Herrera et al. (12), photoperiod imposed a strong and consistent signature on the brain transcriptome where a broad reduction in transcript abundance was found in fish under LL compared to LD. Notably, this global downshift was accompanied by a smaller, functionally cohesive set of transcripts that were significantly upregulated under LL. These upregulated genes were mainly associated with reactive oxygen species (ROS) responses and genome stability, and they also pointed indirectly to growth-related pathways, consistent with the enhanced zootechnical growth reported. This pattern may partly reflect the stringent differential gene expression criteria applied (LogFold change ≥ 2; p-value ≤ 0.05). Although useful for detecting large-magnitude changes, this parameterization can bias interpretation toward differences dominated by transcript copy-number contrasts, potentially overlooking biologically meaningful, coordinated shifts of smaller effect size in directly relevant genes, which is the focus of the present work.

In this study, a major outcome of LL exposure is the remodelling of the somatotropic axis (GH-IGF system), consistent with evidence that extended photoperiods can enhance growth via endocrine mediation in teleosts (22–25). As the primary endocrine regulator of somatic growth in vertebrates (26), this axis is a plausible mechanistic bridge between photoperiod and the observed growth phenotype.

In our data, LL fish showed reduced brain GH transcript levels but increased expression of downstream growth-signaling components, including GHR, IGF2, and IGF1R. This pattern may reflect altered central regulation of brain components of the growth axis, although interpretation in terms of peripheral sensitivity remains speculative without multi-tissue data.

Similar dissociations between GH profiles and IGF signaling have been observed when photoperiod alters endocrine rhythms (25, 27). In contrast, LD fish exhibited features consistent with “classical” rhythmic regulation, in which the 12L:12D cycle supports circadian control of somatostatin and GHRH, thereby shaping GH dynamics (28). Absence of the light-dark cycle under LL may blunt this rhythmic control, promoting a more sustained, less pulsatile regulation of the pathway (29).

Mechanistically, GH acts largely by inducing IGF production (30). In teleosts, IGF-2 remains abundant beyond the embryonic stage, supporting continuous growth and metabolic flexibility across the juvenile and adult phases (31). Elevated GHR and IGF1R under LL could therefore increase tissue responsiveness to available GH, amplifying anabolic signaling even if GH transcription is reduced (32).

Photoperiod-associated differences in somatostatin receptor (SSTR) expression further support divergent regulatory modes between treatments. The diversification of SSTRs in teleosts enables fine-tuned modulation of growth in response to environmental cues (33). Higher receptor expression under LD could operate as negative feedback to stabilize GH secretion under predictable conditions (34). Conversely, reduced expression of some somatostatin receptors under LL may partially disinhibit somatostatin-mediated restraint, and when combined with higher GHR/IGF signaling components, could contribute to sustained growth promotion.

At the level of ligand availability, LL fish showed a concerted downregulation of multiple IGF-binding proteins (IGFBP1, IGFBP2A, IGFBP3, IGFBP5), thereby increasing IGF bioavailability and biasing the endocrine milieu toward growth (35). The exception, IGFBP7, was upregulated under LL. In mammals, IGFBP7 is often described as having relatively low affinity for IGFs, suggesting it may not strongly constrain IGF action (36), although its function in *C. estor* remains unresolved.

The differential expression of inhibitor of growth (ING) family members adds an epigenetic and genome maintenance dimension to photoperiod effects. In mammals, ING proteins act as chromatin regulators influencing histone acetylation and are linked to DNA repair, apoptosis, senescence, and cell-cycle control (37–40). Under LL, upregulation of ING3 and ING4 may reflect an adaptive response to cellular challenges associated with constant illumination, including increased ROS production and circadian disruption, by strengthening DNA repair and chromatin-based transcriptional reprogramming (40). At the same time, ING genes in fish may also intersect with growth regulation, development, reproduction, and modulation of the IGF pathway, suggesting that increased ING3/4 could serve as a compensatory brake to prevent excessive IGF activation in specific contexts (35, 41–44). Under LD, ING2 and ING5 expression may be more consistent with maintenance of homeostasis under predictable rhythmic conditions, though teleost-specific ING functions remain poorly characterized.

Continuous illumination also altered the thyroid hormone axis. Thyroid hormones (T_4_ and T_3_) are major drivers of metabolism, growth, and development in fish (45–47). The higher expression of THRA (thyroid hormone receptor A) under LD and THRB (thyroid hormone receptor B) under LL suggests receptor-specific sensitivity or feedback differences across photoperiods. Although functional distinctions vary across tissues and species, THRB is frequently linked to the growth-promoting and metabolic actions of T_3_ in peripheral tissues, whereas THRA has been associated with developmental timing and cardiac function (46, 48). The concurrent engagement of the thyroid pathway and GH-IGF axis in our transcriptome supports the possibility of synergistic neuroendocrine “push” for growth, since TH can enhance GH secretion and IGF expression, and vice versa (49–51).

Some studies have reported adverse effects of LL in various fish species, particularly when LL is imposed only after an initial developmental period under a 12:12 h light-dark cycle (52, 53). In contrast, in Corona-Herrera et al. (12), fish were reared under LL from fertilization onward, representing a key chronobiological distinction that may partly explain the divergent growth responses and suggests that early-life entrainment to a constant light environment provides a different biological framework in the absence of a day-night cycle. It is important to consider that the present analysis is limited to brain tissue, whereas key components of growth regulation are often predominantly expressed in peripheral organs such as the liver and muscle. Thus, the absence or low expression of certain genes in the brain transcriptome does not preclude their systemic contribution to growth. In this context, the observed transcriptional patterns should be interpreted as reflecting modulation of central components of the growth axis rather than the full endocrine response. Integration with peripheral tissues will be essential to determine whether these changes correspond to shifts in endocrine sensitivity, metabolic state, or broader organismal regulation under continuous illumination.

As previously reported by Corona-Herrera et al. (12), a prominent feature of the LL condition was the upregulation of key antioxidant defense transcripts, which are examined in greater detail here. These included enzymes involved in ROS detoxification, such as superoxide dismutases, glutathione peroxidases, glutathione reductase, and glutathione S-transferases. Similar photoperiod-linked increases in oxidative stress responses have been described under continuous illumination (53, 54), and elevated transcription of detoxification enzymes has also been reported in fish exposed to challenging light regimes (55). Together, these patterns are consistent with the idea that constant illumination imposes a sustained, low-level oxidative challenge that may require antioxidant compensation, potentially coupled to enhanced DNA maintenance and repair processes (55). However, ROS are not exclusively detrimental. When maintained below damaging thresholds, ROS can function as molecules that promote survival and proliferation, forming the basis of hormesis and “oxidative eustress” (56). Under this framework, LL could create a controlled redox environment in which moderate ROS signaling activates growth-supporting pathways (e.g., MAPK/ERK-related cascades) without tipping into oxidative stress (55). The low plasma cortisol levels reported under LL in *C. estor* support the notion that these fish are not in chronic distress but instead may be mounting an adaptive response compatible with sustained growth (12).

An additional, equally plausible and non-mutually exclusive hypothesis is that the fast-growth phenotype itself contributes to the observed ROS signature. Increased growth under LL likely elevates metabolic demand, increasing mitochondrial activity and shifting redox balance. Under this scenario, the differential regulation of ROS-related pathways would primarily result from heightened metabolism rather than a direct photoperiod effect on redox control. The LL-associated upregulation of mitochondrial UCP2 supports the involvement of mitochondrial bioenergetics and oxidative balance in shaping the transcriptomic response. The latter hypothesis requires confirmation.

Expression of the embryonic smooth muscle myosin heavy chain gene (MYSU) provides a complementary view of tissue state. In teleosts, sustained growth and adaptation are supported by high neuronal plasticity, cytoskeletal remodelling, and ongoing neurogenesis (57). Upregulation of MYSU under LD may indicate that natural light-dark cycling supports an active state of plasticity aligned with circadian regulation of proliferation, differentiation, and cytoskeletal remodelling (58). Conversely, downregulation under LL may reflect reduced tissue reorganization processes that depend on intact circadian cues, describing MYSU more as a marker of neuronal plasticity than as a direct growth effector.

Finally, transcriptomic shifts also highlight altered neuroendocrine regulation of appetite under LL. Teleost food intake is regulated by a balance of orexigenic and anorexigenic factors (3), and LL likely disrupts the normal daily cycling of these signals. Fish may compensate by adjusting specific regulators, including somatostatin signaling, which can influence both appetite and growth depending on species and context (59, 60). Reduced expression of some somatostatin receptors under LL could weaken inhibitory tone on feeding centers and GH regulation, potentially facilitating sustained intake and growth (60). This is consistent with observations in aquaculture that extended lighting can prolong feeding opportunities and promote growth when circadian disruption is tolerated (61).

Moreover, the epigenetic marker profile under LL is consistent with dynamic chromatin remodelling biased toward transcriptional repression, while preserving signals compatible with selective transcriptional activation. The upregulation of HDAC4/HDAC7 and KDM5B, together with downregulation of p300, KMT2C, KDM6A, and TAF5L, suggests reduced chromatin permissiveness and may contribute to the broad transcript downregulation previously observed under LL (12, 62–64). Furthermore, the upregulation of these epigenetically repressive genes may play a role in maintaining cellular and genomic homeostasis in LL. For instance, HDAC4/HDAC7 are implicated in synaptic plasticity (65–72). KDM5B, a well-defined transcriptional repressor, also works as a regulator of genome stability by promoting efficient DNA repair (72, 73). Additionally, the downregulated epigenetic genes in LL, p300, KMT2C, and KDM6A could reduce the activation of chromatin marks at enhancers and super-enhancers, impairing the expression of neuron-specific genes and reducing their excitatory identity (72, 74–77). For example, a decrease in p300 activity partially affects the photoreceptor chromatin organization and its gene expression (78).

At the same time, the increased expression of the epigenetic regulators H3.3 and BRD1/BRD3/BRD4 under LL suggests the activation of chromatin remodelling mechanisms associated with a more dynamic transcriptional landscape. The histone variant H3.3 is incorporated into chromatin in a replication-independent manner and is typically enriched in actively transcribed regions, reflecting ongoing nucleosome turnover at specific loci (79–82). In parallel, members of the BRD family act as acetyl-lysine readers, facilitating the recruitment of transcriptional complexes to target genes. Together, these processes are consistent with chromatin remodelling associated with the selective upregulation of antioxidant and growth-related pathways observed under LL, although direct mechanistic links cannot be established from the present data.

Notably, the increased expression of H3.3 in LL may be linked to its accumulation in neuronal and glial chromatin, where it acts as a positive regulator of gene expression required for proper neuronal function and brain plasticity (83, 84). Given that the present analysis was conducted in brain tissue, this finding is particularly relevant. It suggests that continuous photoperiod not only influences endocrine pathways associated with growth but may also modulate epigenetic mechanisms that support neuronal adaptation to prolonged light exposure. In this context, epigenetic regulation may serve as a molecular interface between environmental light cues and the sustained activation of growth-related programs in juvenile *C. estor*.

In addition, the decrease in EZH2 in LL could accelerate photoreceptor differentiation in the postnatal retina, with progressive degeneration and reduced cell death (85, 86). Increased CTCF expression further supports higher-order genome reorganization under LL, potentially strengthening boundaries between compact (repressed) and relaxed (active) chromatin domains (87–89). Besides, CTCF could regulate global chromatin accessibility and transcription during photoreceptor development in LL (82, 83).

Finally, reduced ATM expression indicates an altered DNA-damage-sensing state in LL (90, 91). Whether ATM downregulation reflects reduced genotoxic damage due to effective antioxidant compensation or a rewiring of genome-stability control remains an open question, warranting dedicated assays of DNA damage and repair capacity under LL.

Notably, canonical orexigenic and anorexigenic genes, including NPY and Orexin, were not detected in the brain tissue analyzed in this study (**Table 4**). This finding is consistent with previous observations in *C. estor*, in which these transcripts were also absent from hepatic tissue (92). Together, these results may indicate the presence of an alternative, tissue-specific regulatory mechanism governing appetite in this agastric species with a relatively short and less complex intestine. Further support for this hypothesis would require targeted analysis of gut transcript expression, ideally complemented by a complete genome assembly. However, among detected orexigenic-related transcripts, only the apelin receptor A was upregulated under LL, likely reflecting strong temporal dynamics in appetite gene expression that depend on feeding state and sampling time (3, 93). In parallel, the downregulation of several anorexigenic signals under LL suggests reduced satiety restraint, compatible with increased food intake. However, several appetite-related regulators identified in this study, including spexin and prolactin receptor, are also known to exert significant functions in peripheral tissues in teleosts. Therefore, while the present results primarily reflect central neuroendocrine responses, they likely represent only part of an integrated system regulating whole-body energy balance. Targeted studies in *C. estor* incorporating feeding schedules, time-series sampling, nutritional variables, and multi-tissue transcriptomic and endocrine analyses will be necessary to fully resolve these dynamics and their systemic coordination.

**Figure 1:**
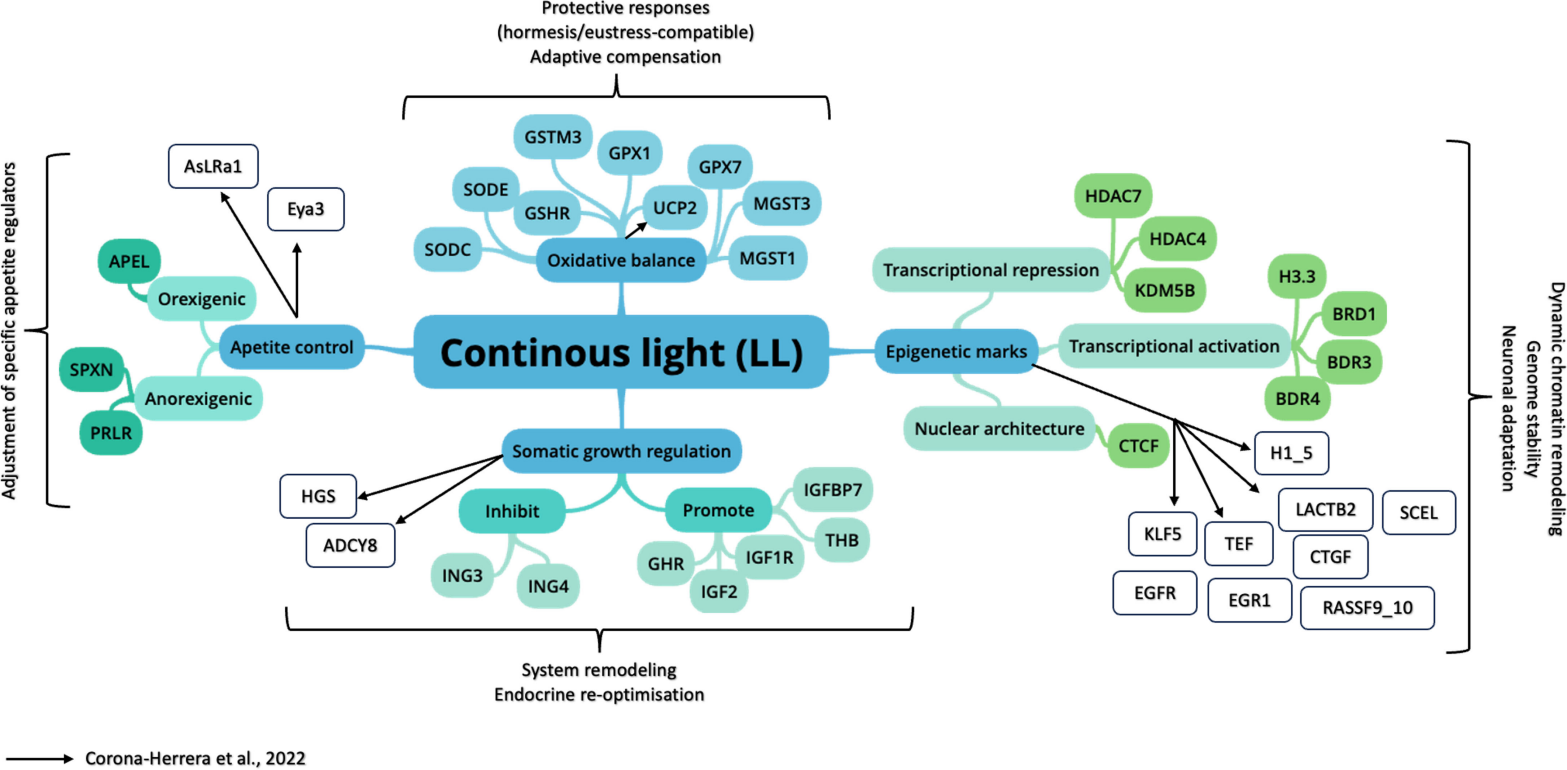
Conceptual overview of genes upregulated under continuous illumination (LL) in juvenile *C. estor*, grouped into four functional categories: somatic growth regulation, oxidative stress (balance), epigenetic marks, and appetite control. Colored connectors indicate association within biological processes in the present study. Solid arrows → indicate genes proposed by Corona-Herrera et al., 2022 (LogFold change ≥ 2 and p-value ≤ 0.05). APEL, Apelin receptor A; SPXN, Spexin; PRLR, Prolactin receptor; HGS, Transformation growth factor; ADCY8, Adenylate Cyclase 8; ING3, Inhibitor of growth protein 3; ING4, Inhibitor of growth protein 4; GHR, Growth hormone receptor; IGF2, Insulin-like growth factor 2; IGF1R, Insulin-like growth factor 1 receptor; THB, Thyroid hormone receptor beta; IGFBP7, Insulin-like growth factor-binding protein 7; CTCF, CCCTC-binding factor; KLF5, Kruppel-like factor 5; EGFR, Epidermal Growth Factor Receptor; TEF, Thyrotrophic embryonic factor EGR1, Early Growth Response 1; RASSF9_10, Ras Association Domain Family; CTGF, Connective Tissue Growth Factor; LACTB2, Beta 2 lactamase; SCEL, Sciellina; H1_5, Binding histone; BDR4, Bromodomain-containing protein 4; BDR3, Bromodomain-containing protein 3; BDR1, Bromodomain-containing protein 1; H3.3, Histone H3.3; KDM5B, Histone demethylase 5B; HDAC4, Histone deacetylase 4; HDAC7, Histone deacetylase 7; MGST1, Microsomal glutathione S-transferase 1; MGST3, Microsomal glutathione S-transferase 3; GPX7, Glutathione peroxidase 7; UCP2, Mitochondrial uncoupling protein 2; GPX1, Glutathione peroxidase 1; GSTM3, Glutathione S-transferase Mu 3; GSHR, Glutathione reductase, mitochondrial; SODE, Extracellular superoxide dismutase; SODC, Superoxide dismutase; Eya3, Eyes absent homolog 3; AsLRa1, Leptin receptor.

Overall, these transcriptomic signatures support substantial physiological plasticity of the “fast growth phenotype” in *C. estor* under LL, further supporting previous suggestions (12) summarized in **Figure 1**. Rather than showing a collapse consistent with chronic stress, LL fish appear to engage protective mechanisms (antioxidant defenses and genome maintenance pathways) while re-optimizing central components (GH/IGF axis, thyroid signaling, and appetite regulators) to sustain accelerated growth. This profile aligns with a hormetic interpretation in which moderate, controllable cellular challenge promotes adaptive responses with net benefits for performance (55), echoing photoperiod-driven growth plasticity reported across teleosts (94–98). Importantly, the integrated alternative hypothesis remains plausible: enhanced growth may elevate metabolic demand and mitochondrial activity, secondarily shaping ROS and redox pathways. Disentangling direct photoperiod effects from metabolism-driven redox consequences will require time-resolved endocrine measurements, mitochondrial functional assays, and circadian-phase sampling, and it also opens a promising line of research into epigenetic regulation of growth in fish.

## 5 Conclusion

The transcriptomic shifts observed (increased ROS defenses, a potentiated GH-IGF-thyroid growth axis, epigenetic remodeling, and altered neuroendocrine appetite control) collectively support the idea that LL prompts a hormetic, beneficial reprogramming of physiological pathways, supporting that strategic photoperiod manipulation can be harnessed (with caution) enabling the fish to achieve good growth and survival rates, exemplifying the remarkable adaptive plasticity of teleost endocrine and metabolic systems in response to environmental changes. *C. estor* offers a model for understanding the interconnection between environmental signals, gene regulation, and growth in teleost fish, facilitating the planning of strategies to improve fish performance in aquaculture.

The transcriptomic patterns observed under continuous illumination in juvenile *C. estor*, including increased expression of antioxidant defenses, changes in brain components of the growth hormone, insulin-like growth factor, and thyroid axes, shifts in epigenetic regulators, and altered neuroendocrine appetite signals, are consistent with a coordinated physiological response to photoperiod. Together, these findings suggest that continuous illumination is associated with a transcriptional state compatible with sustained growth and redox balance, rather than a profile indicative of chronic stress.

It is important to note that this study is based on brain transcriptomic data and, therefore, reflects central neuroendocrine responses rather than the full systemic endocrine and metabolic integration. Future studies that include multiple tissues, endocrine measurements, and functional approaches will be necessary to determine how these transcriptional patterns translate into whole-organism physiology.

Overall, *C. estor* represents a valuable model for understanding how environmental light conditions influence gene regulation and growth-related processes in teleosts, with potential relevance for both ecological research and aquaculture applications.

## Data Availability

The original contributions presented in the study are publicly available. This data can be found here: https://www.ncbi.nlm.nih.gov/search/all/?term=PRJNA401428.
